# Glial Processes at the Drosophila Larval Neuromuscular Junction Match Synaptic Growth

**DOI:** 10.1371/journal.pone.0037876

**Published:** 2012-05-29

**Authors:** Deidre L. Brink, Mary Gilbert, Xiaojun Xie, Lindsay Petley-Ragan, Vanessa J. Auld

**Affiliations:** Department of Zoology, Cell and Developmental Biology, Life Sciences Institute, University of British Columbia, Vancouver, British Columbia, Canada; University of Nebraska Medical Center, United States of America

## Abstract

Glia are integral participants in synaptic physiology, remodeling and maturation from blowflies to humans, yet how glial structure is coordinated with synaptic growth is unknown. To investigate the dynamics of glial development at the Drosophila larval neuromuscular junction (NMJ), we developed a live imaging system to establish the relationship between glia, neuronal boutons, and the muscle subsynaptic reticulum. Using this system we observed processes from two classes of peripheral glia present at the NMJ. Processes from the subperineurial glia formed a blood-nerve barrier around the axon proximal to the first bouton. Processes from the perineurial glial extended beyond the end of the blood-nerve barrier into the NMJ where they contacted synapses and extended across non-synaptic muscle. Growth of the glial processes was coordinated with NMJ growth and synaptic activity. Increasing synaptic size through elevated temperature or the *highwire* mutation increased the extent of glial processes at the NMJ and conversely blocking synaptic activity and size decreased the presence and size of glial processes. We found that elevated temperature was required during embryogenesis in order to increase glial expansion at the nmj. Therefore, in our live imaging system, glial processes at the NMJ are likely indirectly regulated by synaptic changes to ensure the coordinated growth of all components of the tripartite larval NMJ.

## Introduction

Glia are integral parts of synapses in the CNS and PNS of most animals, and regulate many aspects of synaptic development and function. In Drosophila, glia form the blood-brain barrier [1], [2], [3], [4], maintain ion homeostasis [5] and regulate neurotransmitter levels in adult muscles [6], [7]. Glia also remodel growing or injured neurons by engulfment and phagocytosis in the CNS [8], [9], [10], [11]. However, coordination of glial growth and development with neuronal synapses is not well understood.

During development, the Drosophila larval NMJ grows dramatically, and motor synaptic strength adjusts to match muscle input resistance of the growing muscle cells [12], [13]. Increased motor activity as in *Shaker, ether-a-go-go* (*Sh, eag*) mutants [14], [15] and larvae reared at high temperatures (30°C) [16] results in increased motor neuron branching and bouton number. Similarly, dis-inhibition of motor neuron growth and synapse formation, as occurs in *highwire* mutations, results in hyper-expanded neuronal branches and less defined boutons. However, individual *hiw* synapses are less effective than wild type, hence synaptic hypertrophy can occur independently of elevated motor activity [17], [18]. Overall, the conditions and mechanisms that control growth of the pre- and post-synaptic NMJ components have been extensively studied [19], but much less is know about what controls the presence and growth of glial processes to match synaptic growth.

Standard fixation techniques disrupted glial structures at the larval NMJ and so we developed a system to visualize live larval NMJs with all three components, glia, neurons and SSR (subsynaptic reticulum) fluorescently labeled [20]. Of the three glial classes found in the peripheral nerve, we found glial processes from both the perineurial and subperineurial glia at the NMJ. We observed processes from the subperineurial glia (SPG) that formed a septate junction and generated a “blood-nerve barrier” around the motor axon prior to the first proximal bouton. Processes generated by the perineurial glia (PG) interdigitated with the neural bouton terminals and the SSR, and projected onto non-synaptic muscle. These glial processes extended into the synaptic region beyond the end of the blood-nerve barrier but never completely covered the entire NMJ. Glial processes were present at the NMJ as early as the 2^nd^ larval instar and were regulated by conditions and mutations that influenced synaptic structure. We also found that elevated temperature exposure during embryonic development was required for temperature dependent enhancement of the glial processes. Overall the expansion of the glial processes at the larval NMJ appears to be a consequence of regulated growth of the synapse itself.

## Results

### Perineurial Glia Extended Processes into the Live Neuromuscular Junction

**Figure 1:**
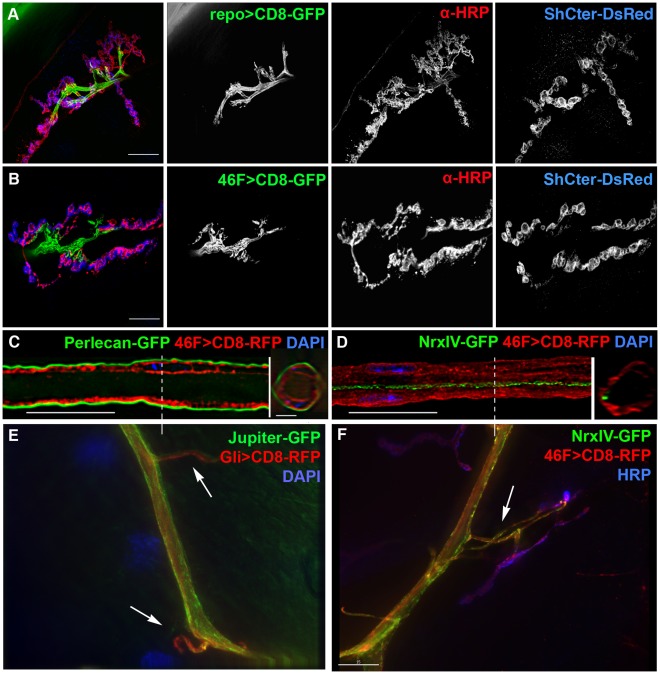
Live imaging revealed that glial processes extended into the NMJ. A, B) Live larval NMJs labeled for all three components of the tripartite synapse. Membrane targeted GFP (CD8-GFP) was expressed in glia using different GAL4 drivers in live NMJs where the axons and boutons were live labeled with an anti-HRP antibody (α-HRP, red) and the SSR labeled with ShCter-DsRed (blue). Each panel is a compressed stack of the entire NMJ (12–14 µm). Scale bars, 15 µm. A) repo-GAL4 driving UAS-CD8-GFP in all glia (repo>CD8-GFP, green) labeled the processes in the NMJ from both the subperineurial and perineurial glia. B) 46F-GAL4 driving UAS-CD8-GFP in only the perineurial glia (46F>CD8-GFP, green) labeled similar glial structures seen with the repo-GAL4 driver. C, D) Membrane targeted RFP was expressed in the perineurial glia using 46F-GAL4 (46F>CD-RFP, red) in relationship with known markers in fixed peripheral nerves. Inserts are cross-sections of the nerve at the points marked by the dashed lines. Scale bars, 15 µm. C) 46F>CD8-RFP (red) in the perineurial glia was adjacent to the secreted neural lamella labeled with Perlecan-GFP (green). The image represents a single 0.2 µm longitudinal section through the center of the nerve. D) 46F>CD8-RFP (red) in the perineurial glia is exterior to the expression of GFP-tagged Neurexin IV (NrxIV-GFP, green) in the underlying subperineurial glia. A projection of the entire stack is shown. E) Jupiter-GFP labeled perineurial glia extend over the subperineurial glia labeled using Gli>CD8-RFP as axons extend from the main nerve to their target muscles (arrows). F) 46F>CD8-RFP labeled perineurial glia extend over the subperineurial glia labeled using NeurexinIV-GFP (Nrx-GFP) as axons (α-HRP, blue) extend from the main nerve to their target muscles (arrow).

To study glia with respect to synaptic development and larval expansion, we developed a live tissue preparation [20]. This preparation consists of live larvae with an intact nervous system, turned inside out, and perfused with an artificial hemolymph containing physiological calcium levels and 5 mM Glutamate to desensitize glutamate receptors and stop muscle contractions [21], [22]. In order to visualize the NMJ in living tissue, we used a combination of tissue-specific promoters to drive expression of fluorescently tagged proteins, coupled with live labeling of neurons with tagged antibodies (Figure 1). The post-synaptic subsynaptic reticulum (SSR) in the muscle was visualized using the C-terminal domain of the Shaker K+ channel fused to DsRed (ShCter-DsRed). This fusion protein was expressed in muscles under the control of the myosin heavy chain promoter. Previous studies have shown that a GFP tagged version of this construct accurately marks the SSR throughout larval stages and is localized to the SSR via an interaction with the MAGUK protein Discs-large [23], [24]. To visualize live neuronal terminals, we bathed the larvae in artificial hemolymph containing a primary antibody against HRP (α-HRP), tagged with either an Alexa 647 or Cy5 fluorophore [20], [25], [26].

Glial membranes were visualized using the GAL/UAS system [27]. Membrane targeted GFP (UAS-CD8GFP) was expressed under the control of a range of glial cell drivers: repo-GAL4, Gli-GAL4 and 46F-GAL4 (Figure 1A, B). Three types of glia are present in the peripheral nervous system: perineurial glia (PG), which are outermost adjacent to the neural lamella; subperineurial glia (SPG), which produce septate junctions and form the blood-nerve barrier; and wrapping glia (WG), which are innermost and ensheath individual axons [3]. Repo-GAL4 drives expression in all three glia [28]. 46F-GAL4 drives expression in the perineurial glia [29], as shown by the close proximity of the membrane marker mCD8 tagged with RFP (46F>CD8-RFP) to the extracellular matrix binding proteoglycan Perlecan, in this case endogenously tagged with GFP (Figure 1C). The subperineurial glia express the septate junction protein NrxIV endogenously tagged wtih GFP (NrxIV-GFP), which lies just beneath the 46F>CD8-RFP layer (Figure 1D). Imaging of living tissue using repo-GAL4 to drive the expression of the membrane marker mCD8 tagged with GFP (repo>CD8-GFP) revealed GFP labeled glial processes that entered the neuromuscular junction and extended over and around both axons and proximal boutons (Figure 1A). Similar processes were observed using actin-tagged with GFP (Figure S1). Previously, subperineurial glial processes were seen extending up the first synaptic bouton [4], [14], [30], [31], [32] or extending small distances into the NMJ [32]. Wrapping glia do not send processes to the NMJ (data not shown). Surprisingly, live imaging of 46F>CD8GFP showed a similar GFP labeling pattern as with repo-GAL4 suggesting that the perineurial glia also send processes into the NMJ (Figure 1B). Under normal rearing conditions (25°C), perineurial processes were present within most NMJs including type Ib; type Is and type II boutons on most body wall and jaw muscles (data not shown). Perineurial glial processes labeled using either 46F>CD8-RFP or Jupiter-GFP (a microtubule associated protein endogenously tagged with GFP) extended over the subperineurial glia (labeled using Gli-GAL4 [30]) and the underlying axons, as they branch off the nerve towards the target muscle (Figure 1E, F).

To test if these perineurial glial processes were under-represented using “standard fixation conditions” used in the field, larvae expressing CD8-GFP driven by 46F-GAL4 were visualized live, or after fixation in a standard saline fixation buffer lacking Ca^2+^. The two-dimensional (2-D) areas of the perineurial processes labeled with GFP and the SSR immunolabeled with anti-Disc-large (Dlg) antibodies were compared. The fixed tissue showed significantly smaller mean 2-D areas in both F3 (7.30+/−3.66 µm^2^, n = 13) and W3 larvae (9.11+/−2.79 µm^2^, n = 14) compared with the GFP labeled processes in live F3 and W3 larvae (11.80+/−3.75 µm^2^ n = 28; and 13.82+/−3.18 µm^2^, n = 20 respectively; P<0.0001) (Table 1). The SSR in the fixed and live tissue had the same mean areas (fixed F3 18.95+/−4.77 µm^2^; fixed W3 18.27+/−3.44; live F3 18.78+/−3.92; live W3 21.01+/−3.92; P  = 0.1690). These results suggest that the extension of perineurial processes into the NMJ were specifically affected by “standard” fixation conditions, which included low extracellular Ca^2+^.

**Table 1:** Statistics for glial, neuronal, SSR areas and ratios.

Category	NMJn =	Larvaen =	Glia: MeanArea +/− S.D.	α-HRP: MeanArea +/− S.D.	SSR: MeanArea +/− S.D.	Glia/HRP	Glia/SSR
WT F3 18°C	24	13	11.71+/−2.93	22.30+/−4.94	20.27+/−4.72		0.59+/−0.14
WT F3 25°C	28	23	11.80+/−3.75	20.97+/−3.59	18.78+/−3.92		0.63+/−0.17
WT F3 30°C	28	19	15.46+/−4.06	24.42+/−6.10	22.49+/−6.10		0.71+/−0.11
Fixed F3 25°C	12	12	7.30+/−3.66		18.95+/−4.77		0.39+/−0.183
Fixed W3 25°C	14	14	9.11+/−2.79		18.27+/−3.44		0.50+/−0.135
*hiw* 18°C	24	13	14.95+/−4.40	27.03+/−5.33		0.55+/−0.12	
*hiw* 25°C	21	11	17.58+/−4.31	29.50+/−4.83		0.60+/−0.13	
*hiw* 30°C	28	21	17.10+/−5.37	27.12+/−4.75		0.65+/−0.24	
TTX 30°C	18	9	10.44+/−3.67		20.39+/−3.25		0.51+/−0.14
WT 3018°C	29	18	13.01+/−4.01		23.35+/−4.28		0.56+/−0.14
WT 1830°C	18	12	11.11+/−2.66		21.10+/−3.76		0.53+/−0.12
W3 25 °C T0′	20	12	13.82+/−3.18		21.01+/−3.92		0.66+/−0.13
W3 25°C T60′	19	12	13.79+/−3.36		21.13+/−4.41		0.66+/−0.12

Mean and standard deviation (SD) values for measured areas of glial processes (CD8-GFP), neurons (anti-HRP immunolabeling) and SSR (ShCter-DsRed). Glial processes were measured from the terminal region of axon as it enters the synapse. The ratios of glial process area to neurons or SSR are indicated. Replicate numbers of NMJs and larvae are indicated for each experimental protocol. F3, feeding third instar larvae. W3, wandering third instar larvae. WT, wild-type. Fixed, fixed larvae; all other larvae were live. T0, T60; 0 and 60 minute time points. 30→18°C, temperature down-shift from 30 to 18°C after hatching. 18→30°C, temperature up-shift from 18 to 30°C after hatching. Areas are in µm^2^.

### The Perineurial Processes Displayed a Range of Interactions with the NMJ

**Figure 2:**
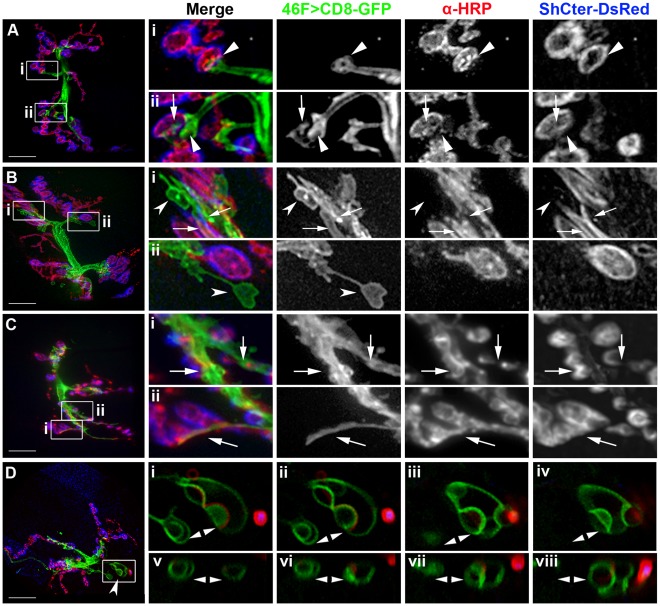
Glial processes at the NMJ had a wide range of morphologies. A–D) Live NMJs with the perineurial glial membranes labeled using 46F>CD8-GFP (green), neurons immunolabeled with anti-HRP (red) and the SSR labeled using ShCter-DsRed (blue). The panels are projections of the entire NMJ (12–14 µm thick) from F3 larvae grown at 25°C. Boxed inserts (i–viii) were digitally scaled 400%. Scale bars, 15 µm. A) A NMJ where the glial processes end with blunt processes at the axon terminus just prior to the first proximal bouton (Ai, Aii; arrowheads). Glial processes also contacted discrete regions of each bouton (Aii; arrows). Glial area  = 13.7 µm^2^. B) A NMJ where the glial processes extended across the muscle surface (Bi, Bii; concave arrowheads) or track along a channel over the bouton and SSR (Bi; arrows). Glial area  = 20.7 µm^2^. C) A W3 NMJ where glial processes had extensive projections into the NMJ and tracked along the center of the synaptic region (Ci, Cii; arrows). Glial area  = 16.85 µm^2^. D) A NMJ where glial processes formed node-like processes embedded in the muscle (concave arrowhead). These processes extended away from the NMJ and were not associated with boutons or SSR. Glial area  = 18.6 µm^2^. D.i–D.iv) Inserts show the node-like glial processes (double arrowheads) embedded in the muscle imaged over a series of single 0.2 µm focal planes. D.v–D.viii) The boxed region in panel D was re-sectioned left to right (v to viii) on the YZ axis. The glial nodes (double arrowheads) were spherical and approximately 3 µm thick. The top of each panel is the approximate coverslip position.

The processes labeled using the perineurial glial driver (46F-GAL4) were examined in greater detail and revealed several distinctive structures. For all our analyses, we concentrated on the NMJs found between muscles 6 and 7 a synaptic region that has been extensively analyzed. First, we observed a cylindrical glial sheath wrapping the motor axons as they extended into the synapse (Figure 2A). These glial sheaths ended in small flared or tapered structures that corresponded to the end of the motor axon branches just proximal to the synapse (Figure 2A; arrowheads). This terminal region of the axon was invariably covered with a glial sheath and observed in 100% of all synapses assayed. Second, we found highly variable lamella-like structures adjacent to and contacting bouton terminals and SSR (Figure 2B; arrows). The glial processes spanned a variable number of boutons but never contacted the entire of bouton arbor or covered the surface of the boutons in a continuous layer. Instead, the glial processes often extended past the first bouton and along the center of the synaptic region (Figure 2B,C: arrows; Video S1). Third, we found lamella-like structures that extended to areas of the muscle that lacked bouton terminals and SSR (Figure 2B: concave arrowheads), occasionally across substantial distances. Lastly, in a small number of profiles (<2%), we observed glial varicosities, apparently three-dimensional nodes, embedded in the muscle (Figure 2D: double arrows). Glial nodes were not associated with either boutons or the SSR in body wall muscle. These last three classes of profiles varied between muscles 6/7 in single larvae and between individual larvae. We found similar morphologies using repo-GAL4 to drive the expression of actin tagged with GFP (Figure S1), hence the GFP- tagged reporters themselves probably did not induce structural variation. Therefore the processes imaged using the 46F-GAL4 driver represent perineurial glial processes present at the larval NMJ, which showed a wide range of morphologies and contacts within regions of the NMJ.

**Figure 3:**
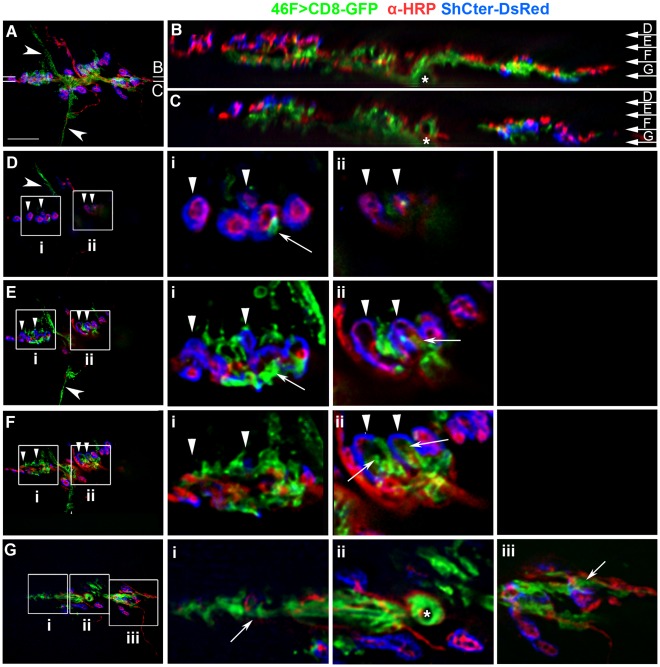
Perineurial glial processes interdigitated with boutons and the SSR. A–F) A live NMJ from a F3 larva raised at 25°C. The perineurial glial processes are labeled with 46F>CD8-GFP (green), the neurons live labeled with anti-HRP (red) and the SSR labeled using ShCter-DsRed (blue). A) A projection of the NMJ (12–14 µm thick). Perineurial glial processes extended through the synapse and beyond the NMJ to contact the muscle surface (concave arrowheads) independently of neuronal or SSR structures. Glial area A = 16.7 µm^2^. Scale bar, 15 µm. B–C) The image stack was rotated at the points indicated in panel A by 90°. The motor axons (asterisk) entered the space between muscle 6 and 7. The GP extended along the base of the NMJ and sent projections up into the overlying bouton/SSR regions. The top (D arrow) represents the surface closest to the viscera, the bottom (G arrow) represents the layers closest to the cuticle and nerve root. D–F) Individual focal planes taken around the points indicated in panels B and C. The boxed regions were digitally scaled 200% and shown to the right (i–iii). D) A superficial focal plane showing the glial processes extending across the muscle (concave arrowhead). A few glial processes extended from below to contact the boutons/SSR (Di, Dii; arrowheads). E) A focal plane where glial processes extended across the muscle (concave arrowhead). At this level the GFP-labeled processes interdigitated with the boutons/SSR and extended between boutons (Ei, Eii; arrowheads) plus contacted the center of the synapse (Ei, Eii; arrows). F) A deeper focal plane within the middle of the NMJ. At this plane the glial processes extended to the bouton layer in the corresponding panels in E (Fi, Fii; arrowheads) or projected along the center of the synapse (Fi, Fii; arrows). G) A deep focal plane at the level of the nerve root entry between muscles 6 and 7. The motor axon entry point and the anti-HRP antibody excluded by the glial sheath are indicated (Gii; asterisk). Glial processes were more expansive at this level and extend along the length of the axons (Gi, Giii; arrows).

We wanted to characterize the details of the perineurial glial processes with respect to the three-dimensional structure of a representative synaptic region from muscles 6/7 (Figure 3; Video S2; Video S3). Starting in the more superficial image focal planes (nearest the viscera), we observed neuronal branches radiating over the muscle surface along with a few flat glial processes that extended away from the synapse over the muscle surface (Figure 3A, D: concave arrowheads). In deeper focal planes, we found neuronal boutons encapsulated by pocket-like SSR, and glial processes interdigitating between these synaptic regions (Figure 3E, F: arrowheads). We observed lamellae-like glial processes that extended down the center of the synaptic region (Figure 3E, F: arrows). However, the glial processes did not cap or completely encapsulate any of the boutons. For muscle 6/7, the motor axons enter between the muscles from the cuticular side and the glial processes were highly concentrated within these deeper focal planes (Figure 3B, C, G: asterisks). Notably, lamella-like processes often appeared in these deeper focal planes near the basal regions of the NMJ (Figure 3G: arrows). Glial processes therefore did not form a simple monolayer covering the NMJ but rather were complex structures interdigitated throughout the perisynaptic channel, and within the space between muscles 6 and 7.

### Only the SPG Create a Blood-nerve Barrier at the Larval NMJ

**Figure 4:**
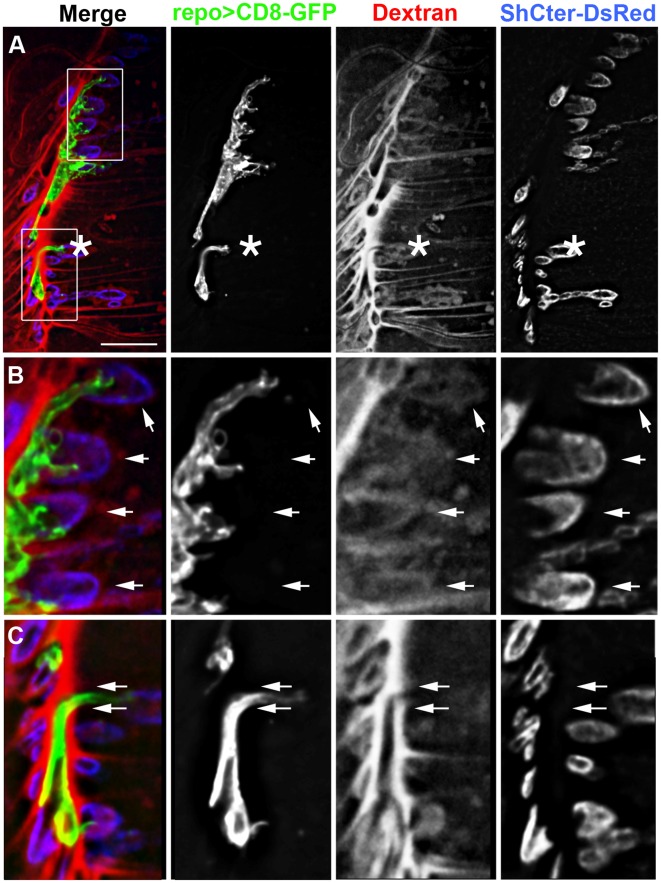
The blood-nerve barrier does not extend beyond the motor axon. A–C) Live NMJ from a F3 larva reared at 30°C. The glial membrane processes were labeled using repo>CD8-GFP (green) and the SSR labeled using ShCter-DsRed (blue). A 10 kDa fluorescent dextran (red) was used to test for the presence of a permeability barrier. Glial area  = 13.77 µm^2^. Scale bar, 15 µm. A) A single Z-slice from a focal plane at the surface of the NMJ showed dextran permeation into the synaptic ^region^. The motor nerve projection is marked (asterisk) between muscles 6 and 7, and showed dye exclusion from the motor axons. B) The boxed region in panel A was digitally scaled by 200%. The fluorescent dextran pooled around the SSR even with the close association of the glial processes (arrows). C) The boxed region in A was digitally scaled by 200%. Fluorescent dextran was excluded along the axon (between the arrows) where the GFP tagged glial membrane was present and demarks the regions where the blood-nerve barrier is found.

One possible function for the glial processes at the NMJ is the formation of a “blood-nerve barrier”. The subperineurial glia (SPG) create a “blood-nerve barrier” along the peripheral nerve through the formation of septate junctions [2], [4], which generate a permeability barrier impermeable to dextrans 10 kDa or greater in size [3], [20], [33]. To test the presence of a permeability barrier at the NMJ, we assessed the ability of a fluorescent 10 kDa dextran dye to diffuse into the synaptic cleft in live preparations (Figure 4). Infused dextran dye reached the surfaces of the body wall muscles and penetrated into the perisynaptic spaces within five minutes of application (Figure 4A). The dye formed oval pools highlighting the perisynaptic space between unlabeled boutons and the SSR, even in the presence of interdigitated glial processes (Figure 4B: arrows). In contrast, glia ensheathing the axons excluded the dextran dye (Figure 4C: arrows). These observations suggest that glial structures at the larval NMJ form two distinct regions: the blood-nerve barrier with associated septate junctions; and the lamella-like glial processes, which extend into the synapse.

**Figure 5:**
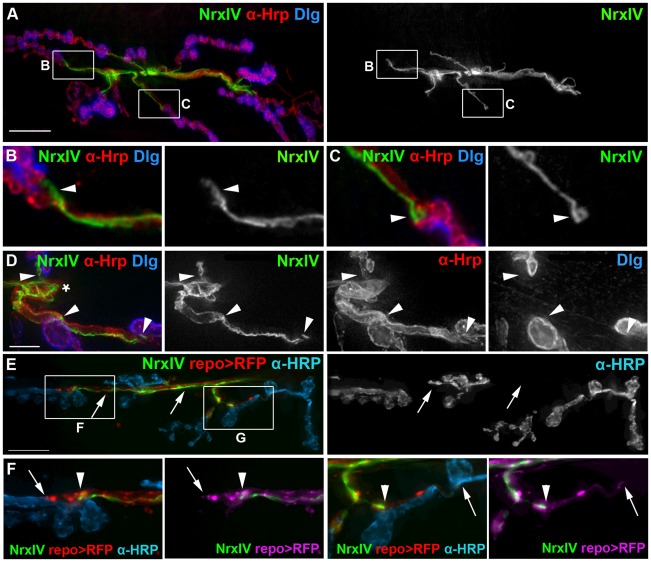
Neurexin IV defined the motor axon end and the septate junction barrier proximal to the synaptic boutons. A–D) Fixed NMJ preparations where the septate junctions generated by the subperineurial glia (SPG) were labeled using Neurexin IV-GFP (NrxIV, green), the boutons and axons with anti-HRP (α-HRP, red) and the post-synaptic SSR with anti-Dlg (Dlg, blue). A 2D projection of the entire stack is shown in each panel. A) A fixed NMJ from a W3 larva with the corresponding grayscale showing the Neurexin IV-GFP. Scale bar, 15 µm. B–C) The boxed regions in panel A were digitally scaled 400% and the corresponding grayscale panels show the Neurexin IV-GFP distribution. Neurexin IV is excluded from synapses; limited to small axon branches and terminates prior to the first bouton. The septate junction termini formed blunt or tapered ends (B; arrowhead) with the occasion bulb-like structure (C; arrowhead). D) A fixed NMJ from a F3 larva in which the panels have been digitally scaled 300%. The septate junction continued along the axon from the root (asterisk) but stopped just before the proximal bouton of each branch (arrowheads). Scale bar, 5 µm. E–F) A live NMJ from a F3 larvae with the septate junctions labeled with Neurexin IV-GFP, the glia labeled using repo>CD8-RFP (red) and the neurons/boutons lived labeled using anti-HRP antibodies (blue). E) Fluorescently tagged anti-HRP antibody (α-HRP, blue) labeled the NMJ including those areas contacted by glial processes (repo>RFP, red) but the axons remain unlabeled (arrows) in the regions bounded by the septate junctions (NrxIV, green). The grayscale image shows the extent of anti-HRP antibody immunolabeling and the unlabeled axons (arrows). F, G) The boxed regions in panel E were digitally scaled 200%. Glial processes (arrows) labeled with RFP (repo>RFP) (I, J; red: Ii, Ji; magenta) extend beyond the terminus of the septate junction (NrxIV, green) (arrowheads).

Using repo-GAL4, Gliotactin-GAL4 and 46F-GAL4, we observed glial processes that extended beyond the septate junction into the synaptic region. To investigate the relationship between the blood-nerve barrier and the glial processes that extend into the synaptic region, we used Neurexin IV endogenously tagged with GFP (NrxIV-GFP) to visualize the septate junctions [2], [3], [4]. In both living and fixed tissues, NrxIV-GFP terminated on small axon branches proximal to the first synaptic boutons (Figure 5; Video S4). NrxIV-GFP was not located near the SSR immunolabeled with antibodies to Discs-large (Dlg) (Figure 5A–D) or with the boutons immunolabeled with anti-HRP (Figure 5A–G). The terminal ends of the septate junctions formed tapered lines (Figure 5B; arrowhead), blunt endings (Figure 5D: arrowhead) or bulb-like structures (Figure 5C: arrowhead). The NrxIV-GFP distribution suggested that the septate junctions form a permeability barrier at the motor axon end just prior to the first synaptic bouton. To functionally test for the presence of a diffusion barrier, we labeled live larval preparations expressing NrxIV-GFP with a fluorescently tagged primary antibody against HRP (Figure 5E–G). The antibody labeling was absent from the regions bounded by NrxIV-GFP (Figure 5F, G: arrows) and commenced distal to the NrxIV-GFP terminus (Figure 5F, G: arrowheads), confirming the antibody was excluded from glial structures expressing NrxIV. In contrast, the glial processes that extend into the NMJ did not exclude the anti-HRP antibody from labeling nearby boutons (Figure 5F, G: arrows). These results indicate that barrier function terminates at the septate junction termini labeled with NrxIV and is not a general property of glial processes within the NMJ proper.

### Expanded NMJ Structure Correlates with Increased Frequency and Size of Glial Processes

During development, the larval NMJ must expand its size and strength in order to match the increasing size and input resistance of innervated muscles. In concert, the glial processes at the NMJ should match this expansion to ensure the integrity of the blood-nerve barrier and other functions. NMJs can be induced to increase size, bouton number and neuronal branching frequency by increasing rearing temperature, synaptic activity, or altering signaling pathways [34]. We wondered if the growth of the glial processes is coordinated with that of the NMJ and thus would be regulated by similar factors.

Chronically rearing larvae at 30°C significantly increased the number of axon terminal branches and boutons of the larval NMJ [16], [35]. Therefore, we examined the impact temperature had on both the frequency, and size, of the synaptic glial processes. To measure glial processes we used the membrane CD8-GFP marker and assayed for 2-D areas of GFP projections at the synaptic regions of muscle 6/7. For 2-D analysis the glial sheath around the nerve entering the synaptic junction was excluded. We limited out measurements to the lamella-like process and the small sections of glia that cover the motor axon branches within the synapse. We also scored synapses as +/− for glial processes distal to the first synaptic bouton, as complementary test for frequency of glial projections into the synaptic region.

**Figure 6:**
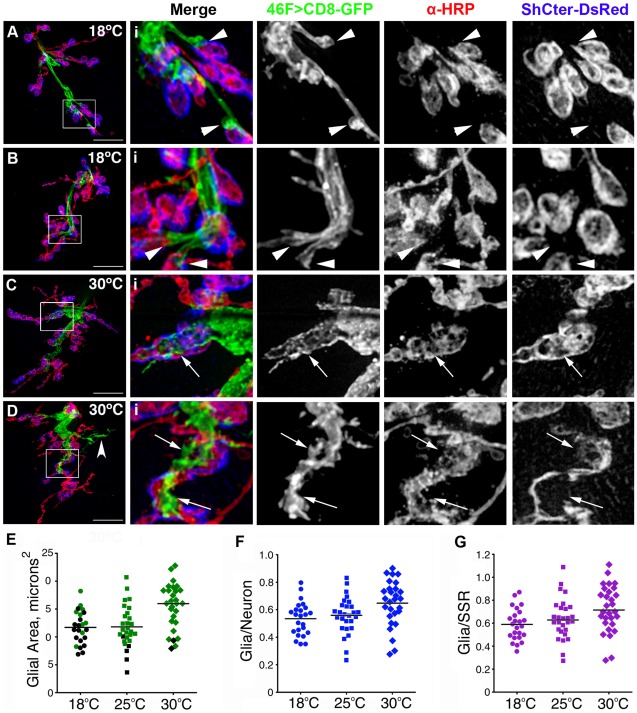
Increased rearing temperature expanded the perineurial glial processes. A–D) Live NMJs from F3 larvae with glial processes labeled using 46F>CD8-GFP (green), axons plus boutons immunolabeled with anti-HRP (red) and the SSR labeled with ShCter-DsRed (blue). Scale bars, 15 µm. The boxed regions (i) were digitally scaled 400% and all three channels shown in grayscale. A–B) NMJs from larvae raised at 18°C. Ai–Bi) Glial processes were small and most stopped at the axon end prior to the proximal boutons in the region corresponding to the end of the blood-nerve barrier (arrowheads). Glial areas: A = 15.7 µm^2^; B = 14.0 µm^2^ C–D) NMJs from larvae grown at 30°C. Ci–Di) Glial processes were more extensive and processes occupied a central channel along the synapse (arrows), plus extended across the muscle away from the NMJ (concave arrowhead). Glial areas: C = 16.0 µm^2^; D = 20.1 µm^2^ E–G) Glial membrane areas were measured and compared to neuron/bouton area (anti-HRP) and SSR area at 18°C, 25°C and 30°C. E) The mean area of glial processes (green) was significantly higher in larvae reared at 30°C (diamonds) than 18°C (circles) or 25°C (squares) (P<0.0002). Black symbols represent NMJs with glial processes that stopped at the motor axon end prior to the first bouton in the region defined as the terminus of the blood-nerve barrier. F) The mean ratio of glial process (GP) to neuron/bouton area (anti-HRP)(blue) was significantly greater at 30°C (diamonds) compared to 18°C (circles) and 25°C (squares) (P<0.0125). G) The mean ratio of GP to SSR area (magenta) was significantly greater for larvae reared at 30°C (diamonds) compared to 18°C (circles) and 25°C (squares) (P<0.0302).

To test if glial processes at the NMJ responded to elevated temperature, we compared glial processes from feeding third instar larvae reared at three temperatures: 18, 25, and 30°C. We found that the GFP labeled processes were larger and had a higher frequency of expansion into synaptic regions at elevated temperatures (Figure 6). In addition, the type of glial processes qualitatively changed with elevated temperature. Profiles most prevalent at 18°C were blunt, shortened and terminated at the end of the motor axon in the region defined by the septate junction terminus (Figure 6A, B: arrowheads). At elevated temperatures glial processes extended further into the synapse, with more frequent processes in the synaptic region (Figure 6C, D: arrows). Occasionally the glial processes extended across the muscle, independent from the labeled boutons and SSR (Figure 6D: concave arrowhead).

When these differences were quantified (Table 1), at 30°C the mean area of the glial processes (15.46+/−4.06 µm^2^; n = 18) was significantly larger than at 18°C (11.71+/−2.93 µm^2^ n = 24) or 25°C (11.71+/−2.93 µm^2^, n = 28, P<0.0002)(Figure 6E). Chronic rearing at 30°C also significantly enhanced the frequency of glial processes within the synapse relative to 18°C (P<0.0001). In our studies, the mean area of the SSR (measured with ShCter-DsRed) showed a small but significant increase between 25°C (18.78+/−3.92 µm^2^) and 30°C (22.49+/−6.10 µm^2^, P<0.0235). Overall we observed glia/SSR ratios that were significantly larger at 30°C (0.72+/−0.11 µm^2^) compared with 18°C rearing (0.59+/−0.14 µm^2^,P<0.0302). Temperature therefore had a strong influence on glial process size, whether the processes extended into the synapses, and how they physically interacted with the synaptic structures.

**Figure 7:**
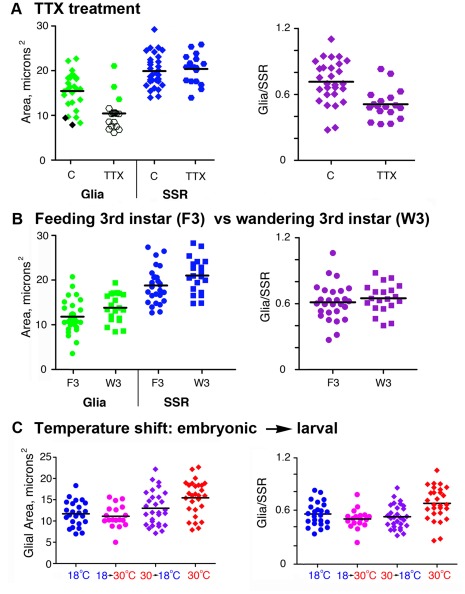
Changes in motor activity and synaptic growth affect the presence of glial processes at the larval NMJ. Live NMJs from F3 larvae with glial processes labeled using repo>CD8GFP, neurons immunolabeled with anti-HRP and the SSR labeled with ShCter-DsRed. A) TTX treatment: comparison of glial process areas (green) and SSR areas (blue) from control (C, diamonds) or TTX treated (TTX, hexagons) larvae raised at 30°C. TTX significantly reduced the mean area of glial processes relative to control animals (P<0.0001) but had no effect on the mean SSR area. Black symbols represent NMJs with glial processes that stopped at the motor axon end just prior to the first bouton. The mean ratio of glial process to SSR area (magenta) was significantly reduced in TTX treated larvae (hexagons) compared to control larvae (diamonds) (P<0.0004). B) F3 compared to W3: comparison of glial (green) and SSR areas (blue) from feeding third instar (F3, circles) or wandering third instar (W3, squares) larvae raised at 25°C. Mean glial areas and mean SSR areas were not significantly different between F3 and W3. The mean ratio of glial to SSR area (magenta) in F3 larvae (circles) was comparable to the ratio in W3 larvae (squares). C) Temperature shift treatment: comparison of glial areas from larvae reared continuously at 18°C (blue circles), shifted from 18°C to 30°C (pink circles), shifted from 30°C to 18°C (purple diamonds) and raised continuously at 30°C (red diamonds). The mean glial area from larvae transferred from 18°C to 30°C was significantly smaller than in larvae grown continuously at 30°C (P<0.0002), but the same size as larvae grown at 18°C. The mean ratio of glia to SSR area at 30°C constant rearing temperature was significantly different from all other protocols (P<0.002).

To test the effect of blocking motor activity on the development of glia at the NMJ, we reared larvae on a diet of TTX at a concentration known to impair foraging behavior without killing the larvae [36]. We analyzed larvae at 30°C to ensure the maximal projection of glial processes and found that TTX treatment caused a reduction in the frequency and the size of glial processes at the NMJ. The effect of TTX was quantified (Table 1) and the mean area of the glial processes was significantly reduced by chronic TTX treatment (10.44+/−3.67 µm^2^ TTX, n = 18 vs. 15.46+/−4.06 µm^2^ untreated, n = 28, P<0.0001) (Figure 7A). In particular TTX reduced the frequency that glial processes entered into the synaptic regions compared to controls (P<0.0001). Since the mean SSR area was not significantly reduced (20.39+/−3.25 µm^2^ TTX vs. 22.49+/−6.10 µm^2^ untreated) the ratio of GP to SSR area was smaller in TTX treated animals (0.51+/−0.14 µm^2^ TTX vs. 0.72+/−0.11 µm^2^ untreated, P<0.0004) (Figure 7A). In other words, reduced motor activity blocked the both the increase in the size, and the likelihood of glial processes being present at the NMJ.

Before metamorphosis, fly larvae at the feeding 3rd instar stage (F3) change behaviors from food foraging and growth to pre-pupal wandering (stage W3). Wandering is a period in which larvae cease feeding and engage in directed migration away from food sources with increased motor activity [38]. Neuronal bouton and arbor number stabilizes prior to F3 stage [16], [24], [37], but bouton size increases in concert with increased motor output [38]. We tested the idea that the increased endogenous motor activity in W3 larvae had a similar effect on glia by visualizing changes to GFP labeled processes. Overall when we assayed W3 larvae there was no significant increase in the mean area of the glial processes (13.82+/−3.18 µm^2^; P  = 0.0564) (Figure 7B), the mean SSR area (21.01+/−3.92 µm^2^ ) or the mean glia/SSR area ratio (0.67+/−0.13, P = 0.4827) (Figure 7B) compared to F3 larvae (0.63+/−0.17).

We also wanted to test for localized changes in glial processes by visualizing changes over set timetime. For these observations, we assumed that endogenous motor activity in our *in situ* preps was similar to early W3 larvae *in vivo*. With our protocol, we observed spontaneous rhythmic contractions in early W3 larvae (n = 5) inverted inside-out in artificial hemolymph (HL-6) containing physiological Ca^2+^ (data not shown). Images captured from W3 larvae at two time points, an hour apart, showed only small, highly localized changes in the morphology of glial processes and SSR (Figure S2). We observed some glial process expansion within the synapse and across the muscle surface independent of boutons and SSR but also observed processes that retracted or were unchanged over the 60-minute period. Overall over one hour, we found no significant change in either the mean glial process area (T0 = 13.82+/−3.18 µm^2^ vs. T60 13.79+/−3.36 µm^2^; P = 0.911) or SSR area (T0 = 21.01+/−3.92 µm^2^ vs. T60 13.79+/−3.36 µm^2^, n = 19; P = 0.5335) (Table 1).

Our *in situ* data suggest that the glial and SSR morphology of the NMJ are stabilized in early W3 larvae, even in the presence of elevated endogenous motor activity.

### Glial Processes Grow in Conjunction with NMJ Growth

**Figure 8:**
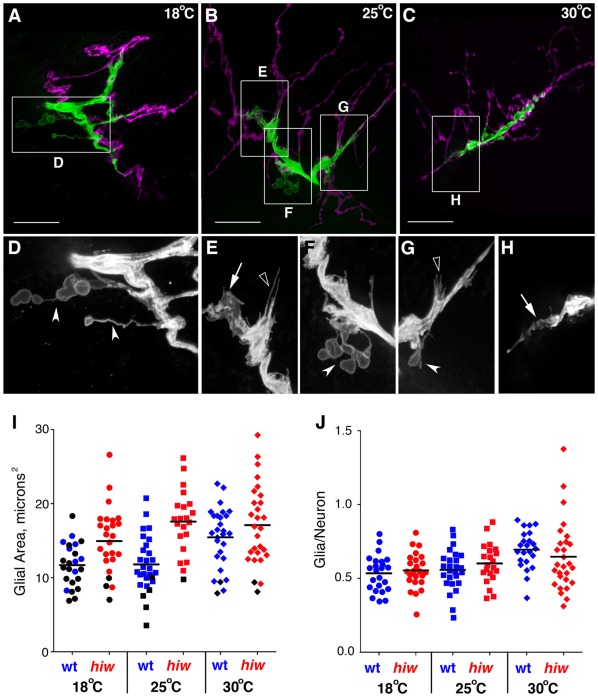
Glial processes were expanded in the highwire mutation. A–C) Live NMJs from F3 larvae with glial processes labeled with repo>CD8GFP (green) and boutons immunolabeled with α-HRP (magenta). NMJs are shown from *hiw* larvae raised at 18°C (A), 25°C (B) and 30°C (C). Glial areas: A  = 26.7 µm^2^; B  = 26.2 µm^2^; C  = 16.7 µm^2^. Scale bars, 15 µm. D–H) Grayscale images showing details of the glial processes from the corresponding boxed regions in A–C. Panels were digitally scaled 200%. Glial processes extended away from the NMJ across the muscle surface (arrowheads) or tracked along the center of the bouton area (arrows). Filopodial-like processes were also frequently observed (open arrowheads). I–J) Statistical comparison of the glial process areas from F3 *hiw* larvae (*hiw*; red) compared to wild-type (wt; blue) larvae raised at 18°C (circles), 25°C (squares) or 30°C (diamonds). I) The mean area of glial processes was significantly larger in *hiw* mutants than wild-type at 18°C and 25°C (P<0.0001) but not 30°C. Black symbols represent those NMJs with glial processes that stopped at the motor axon end. J) The mean ratio of the GP area to neuronal (α-HRP) area was not significantly different between *hiw* and wild-type larvae at any rearing temperature.

Increased temperature affected the presence of glial processes at the NMJ but these effects could be due to factors such as elevated motor activity or structural changes associated with growth of the synapse [15], [36]. To test this possibility, we analyzed the effect of the *highwire* (*hiw*) mutant on glial processes within the NMJ. Highwire dis-inhibits synaptic growth and generates terminals that are expanded, highly branched, and have less defined bouton varicosities [18], [39]. Furthermore, *hiw* mutant synapses have impaired synaptic function, with low quantal content and reduced spontaneous and evoked transmitter release [17]. We observed the NMJs from homozygous *hiw* larvae raised at 18, 25 and 30°C consistently had hyper-expanded branches with less defined boutons (18°C: mean areas 27.03+/−5.33 µm^2^, n = 24; 25°C: 29.50+/−4.83 µm^2^, n = 21; 30°C: 27.12+/−4.75 µm^2^, n = 28) (Figure 8A–C). Glial processes at the *hiw* mutant NMJs had extensive lamella-like and filopodial-like projections along the branches (Figure 8E, G: open arrowheads). In addition we observed an increase in the number of glial processes that extended away from the synaptic region and displayed node-like varicosities across the muscle (Figure 8D, F: concave arrowheads; Video S5). Overall the *hiw* mutants had glial processes that appeared similar to those seen in wild-type larvae raised at 30°C.

When quantified (Table 1), the mean area of glial processes in *hiw* mutants were not significantly different amongst all temperatures tested (18°C: 14.95+/−4.40 µm^2^; 25°C: 29.50+/−4.83 µm^2^; 30°C: 27.12+/−4.75 µm^2^; P  = 0.1391) (Table 1). Essentially, the *hiw* mutation resulted in the growth of large, elaborate glial processes into the NMJ at all temperatures. Therefore the mean areas of glial processes in *hiw* mutant larvae were significantly larger than wild-type at 18°C (P<0.05) and 25°C (P<0.001) (Figure 8D). Similar to elevated temperature, *hiw* mutants also increased the likelihood of glial processes being present in the synaptic region at 18°C compared to wild-type (P<0.0003).

*Hiw* is an E3 ubiquitin ligase known to regulate both synaptic physiology and synaptic growth, by controlling the activity of regulatory pathways such as BMP [18], [39], [40]. We tested for involvement of the BMP signaling in mediating glial growth at the NMJ but were unable to detect the presence of phospho-MAD, a target of the BMP pathway, in any of the three classes of peripheral glia (data not shown). This was not unexpected given that Hiw functions within motor axons and mutants can be rescued with neuronal expression [18]. Therefore the expansion in the glial processes observed in the *hiw* mutants is likely an indirect response to the expansion of the NMJ itself.

### The Presence of Glial Processes at the NMJ is Determined in Embryonic Stages

Previous studies using fixed tissue suggested that glial processes do not extend to the NMJ until late first or early second instar stages [30]. In addition perineurial glia are not observed wrapping peripheral nerves in late embryos or early larval stages [41]. We were able to detect glial processes at the NMJ of 2nd instar larvae using either the perineurial glial driver 46F-GAL4 (Figure S3A–D) or the pan glial driver repo-GAL4 (Figure S3E, F).

Given that perineurial glia appear later in larval development, we tested for a critical developmental period in which we could promote glial process expansion at the NMJ. We used the strong enhancing effect of high temperature on glial processes in bi-phasic rearing protocols. Specifically embryos were developed at 30°C and then switched after hatching to continue larval development at 18°C (“down-shifted”); or embryos were raised 18°C and then switched to 30°C (“up-shifted”). Surprisingly glial processes in “up-shifted” larvae had simple morphologies characteristic of larvae raised continuously at 18°C. “Down-shifted” larvae had complex and expansive glia processes similar to larvae raised constantly at 30°C.

When these differences were quantified (Table 1), the mean area of the glial processes from “up-shifted” larvae was similar to those reared constantly at 18°C (11.11+/−2.66 µm^2^, n = 18, vs. 11.71+/−2.93 µm^2^, n = 24; P = 0.5033) but significantly smaller than larvae raised constantly at 30°C (11.11+/−2.66 µm^2^, n = 18 vs. 15.46+/−4.06 µm^2^, n = 28; P<0.0002) (Figure 7C). In contrast, glial processes in “down-shifted” larvae had a mean area (13.01+/−4.01 µm^2^, n = 29) that was intermediate between those raised continuously in either 18°C or 30°C but was not significantly different from either (P  = 0.1900 and P>0.05 respectively) (Figure 7C). Thus the temperature of embryonic rearing determined the size of the glial processes through further phases of larval life. The timing of the temperature shift had a similar effect on whether or not glial processes were present in the synapse. Glial processes in “down-shifted” larvae (transitioning from 30° to 18°C) were just as likely to be present as those raised continuously at 30°C (P  = 0.1468) and significantly more prevalent than in larvae raised exclusively at 18°C (P<0.0022). Glial processes in “up-shifted” larvae (18°C to 30°C) were less frequent compared to those reared at 30°C (P<0.01) and were similar to those continuously reared at 18°C (P = 0.5178). Overall these findings suggest that elevated temperature exposure during embryonic development predisposed the later growth of large glial processes at the NMJ.

## Discussion

Using the living imaging system developed to visualize all three components present at the neuromuscular junction, we tested the developmental regulation of glial processes in concert with synaptic expansion. The extension of processes from the subperineurial glia (SPG) into the NMJ has been observed previously [4], [14], [30], [31], [32]. We determined that the SPG create the blood-nerve barrier at the axon terminus just proximal to the first synaptic bouton. TEM analysis has shown that motor axons entering the synapse are ensheathed by a glial wrap that is bounded by septate junctions [14], [42]. We observed this same region labeled by a thin band of NrxIV-GFP. This region was stable and was observed in both live and tissue fixed using standard techniques. The stability of this region, and the continued presence of a glial sheath covering the motor axon just prior to first bouton, suggests that the SJ domain stabilizes the glial processes in this region.

Conversely SPG and, surprisingly, perineurial glia (PG) also extend processes further into the larval NMJ. These processes were sensitive to a standard fixation protocol with low extracellular Calcium., While glial processes are found at the NMJ in a wide range of animals, the structure of the glial processes at the Drosophila larval NMJ differed from vertebrate perisynaptic Schwann cells in that they did not cap the synaptic boutons or match the full extent of the synaptic bouton arbors. Instead, live tissue imaging revealed that the perineurial processes extended into deeper focal planes below the surface of the synapse and interdigitated with the synaptic boutons. Furthermore, the perineurial processes extended substantial distances over non-synaptic areas of the muscle surface. The lack of extracellular Ca^2+^ in the fixation solution may have affected the glial morphology, suggesting that glial projections into the synaptic regions are mediated by Ca^+2^ adhesion mechanism. However at this point the identity of the adhesion protein or complex is unknown as neither classic cadherins or integrin subunits could be found associated with glial processes (data not shown).

Our analysis also determined that the extent and the frequency of the glial processes within the synaptic region were tied to changes to the synapse. While we observed a decreased glial presence and area at the NMJ in response to decreased activity, as in our TTX fed larvae, overall our results suggest that the glial processes respond to changes in synaptic structure and size. In particular our results using the *highwire* (*hiw*) mutation support this conclusion. In *hiw* larvae, the NMJ is greatly expanded due to a dis-inhibition of synaptic growth but individual *hiw* bouton synapses have reduced activity [17]. Therefore it appears that glial processes expanded in response to increased synaptic growth rather than an elevation in synaptic activity. Alternatively the reduction of local synaptic activity in *hiw* mutants might lead to an expansion of the glial processes.

Our results suggested that the growth of glial processes into the synaptic region is coordinated with changes that occur to the NMJ as a whole. Physiologically this would ensure that the presence of the blood-brain barrier created by the SPG is maintained through out the expansion of the larval NMJ and any changes to the synaptic size would be matched by a related change in the SPG processes. The role of the SPG glia in forming the blood-nerve barrier is well established and is critical to maintain the physiological integrity of the larval nervous system [2], [3], [4]. We confirmed the presence of a blood-nerve barrier that was limited to the axon branches and correlated with the septate junction protein Neurexin IV. This finding extended previous observations on diffusion boundaries formed by peripheral nerves [3], [14] and matches recent TEM data showing SJs along the glial processes at the place were the axon branch enters the NMJ [42]. In contrast, the remainder of the NMJ, was open to rapid diffusion of large molecules into the perisynaptic space, even in the presence of extensive contact by glial processes.

Perineurial glia form the outer glial sheath of the peripheral nerves in larvae and are thought to be analogous to the mammalian perineurium of peripheral nerves [43]. However perineurial glia arise later in larval development and are not found at embryonic or even early larval stages [44] and as yet their role has not be determined. Both SPG and perineurial glial processes extend into the synaptic region beyond the SJ barrier. This region is characterized by actin rich processes, another feature of the NMJ glia potentially sensitive to fixation conditions. However the functional role of the glial processes and the mechanisms that underlie glial contact have yet to be determined.

One proposed function for the glial processes within the NMJ is phagocytosis, specifically of boutons, during synaptic development and remodeling. Glia engage in phagocytosis in many circumstances during Drosophila nervous system development [8], [10], [45], including engulfment and removal of “ghost-boutons” from the NMJ [32]. However, we could not detect evidence for glial processes engaged in phagocytosis under our experimental conditions. This was even after the temperature “down-shifts” from 30°C to 18°C or TTX treatment, which reduced the size of the glia processes at the NMJ. We did observe a small percentage of glial varicosities or nodes apparently embedded in the muscle but these were often some distance away from the synaptic region and independent of neuronal or SSR labeling. Hence the glial processes were not likely phagocytic. We did not assay for glial endocytosis in 2nd instar larvae so it is possible that glial-based phagocytosis may operate at earlier stages. However, our findings suggest that the glial processes were not correlated with a low number of synaptic boutons, since glial processes were minimal in larvae reared at 18°C, which have reduced numbers of synaptic boutons [16]. Lastly, other data indicate that bouton endocytosis is more likely a function of muscle or neuronal autophagy [32], [46].

Another potential role for the glial processes at the NMJ could involve an association with trachea. In the adult DLM neuromuscular synapse, glial processes project beyond their axonal contacts and often interact physically i with trachea. Trachea are responsible for gas exchange and in the larvae fine tracheal processes around found across the muscles surface. This has lead to a proposal where glial processes couple trachea and presynaptic boutons and function to regulate gas exchange [7].

In our study, TTX decreased the area of glial extensions at the synapse, relative to control larvae, suggesting that TTX based activity reduction had some regulatory effect on glial processes at the nmj. However, the total area of the SSR was comparable between drug-treated and control populations. Thus, reduced glial presence did not stem from more generalized effects of decreased motor activity, for example compromised nutrition. While our results point to an indirect role of neuronal activity on glial process growth, activity dependent regulation of glial development and differentiation is well documented. In vertebrates glial development is intricately tied to neuronal activity such that loss of action potentials due to TTX treatment blocks oligodendrocyte proliferation and myelination [47], [48]. Neuronal regulation of glial cell development has been linked to axonal neurotransmitter release including glutamate which in turn evokes increased glial [Ca^2+^]_i_ in an activity-dependent manner [49], [50]. Similarly ATP release from axons can affect Schwann cell, astrocyte or oligodendrocyte development [51], [52], [53]. At the vertebrate NMJ, motor nerve stimulation induces similar intracellular Ca^2+^ increases in perisynaptic Schwann cells through the release of neurotransmitters, acetylcholine and ATP [54], [55]. Furthermore these responses can be potentiated by neurotrophins NT-3 and BDNF [56]. Changes in synaptic activity also modulate the glial cell cytoskeleton in the perisynaptic Schwann cells [57]. Beyond axon to glia signaling, it is also possible that the SPG are signaling to the overlying perineurial glia. In the peripheral nerve there is evidence of signaling from the SPG to the perineurial layer through Ras-PI3K-Akt activity to promote growth of the perineurial glia [43] as well as neuropeptides or neurotransmitters have been implicated in controlling perineurial glial thickness [58].

If the glial processes are responding to changes in synaptic structure or developmental cues, then when are these cues set during development? We could only detect perineurial glia at the NMJ starting in the second instar stage. Yet we found that the rearing temperature during embryogenesis played a critical role in the determining the extent of glial processes in the 3rd instar NMJ. Intriguingly, embryos grown at 18°C and then up-shifted to 30°C at hatching had very small glial processes in F3 larvae, similar to those in larvae grown continuously at 18°C. This was even with the enhanced locomotion and motor activity that would occur throughout the larval instar stages. These results further support the conclusion that glial processes are not responding to changes in motor or synaptic activity alone but rather are responding to changes in structural or molecular constituents of the NMJ set during embryonic development.

## Materials and Methods

### Fly Strains

We used repo-GAL4 [30], 46F-GAL4 (DGRC, Kyoto), and Gli-GAL4 (Sepp et al., 2000) to drive expression in all glia, perineurial glia or subperineurial glia respectively. UAS-CD8GFP [59] and Neurexin IV-GFP [3] were obtained from the Bloomington stock center. Dr. Elizabeth Gavis donated UAS-CD8-RFP; and *hiw* [DN8] was obtained from Dr. Haghighi. To label the SSR of the neuromuscular junction, we modified a CD8GFP-ShCter construct [24]. The GFP tag was replaced with DsRed by excision of the GFP insertion in pKZ2 (obtained from Dr. C. Goodman) using Spe1. DsRed was amplified with in-frame Spe1 restriction sites from the pRedH-Pelican vector using a Pel5 primer (5′ ACTAGTGCCTCCTCCGAGGACG 3′) and a Pel3 primer (5′ ACTAGTCAGGAACAGGTGGTGGC 3′), and subcloned into pGEMT-1 (DsRed-pGEMT). The DsRed fragment was isolated using Spe1 and introduced into the pKZ2 vector to generate pKZ2-DsRed. Transgenic flies were generated using standard approaches (Genetics Services) and multiple independent insertions generated. Lines were verified for signal strength and specific expression of CD8-DsRed at the SSR. Two lines, BJ4 and BJ10, were used here because of their strong fluorescence.

### Fly Rearing and Staging

Larvae were raised at low density on yeast supplemented potato media and reared at 25°C, or as specified in continuous temperature exposure at either 18°C; and between 29°C and 30°C (denoted as 30°C). We also reared larvae with biphasic temperature exposures, transferring animals from 18°C to 30°C (and the converse) immediately on hatching. We staged feeding third (F3) instar larvae based on size, spiracle morphology, and foraging behavior in the food; and wandering third (W3) larvae were staged by tufted, non-everted spiracles and active upward crawling behavior.

### Pharmacology

For down regulation of larval motor activity at 30°C, we added TTX (Tocris) to instant fly media (Carolina Biological Supply) at a concentration of 0.1 mg/g wet food [36], with larvae transferred daily to fresh TTX supplemented media. Only live, actively foraging larvae were selected for observation.

### Live Tissue Preparation for Imaging

The tissue preparation for live imaging was essentially as described previously [20]. Briefly, we required a tissue preparation that featured relaxed body wall muscle at rest length, and achieved this in preparations of everted live larvae. In all live image studies, the tissue was perfused (about 2 ml/hr) with HL-6 containing 2 mM Ca^2+^ and supplemented with 5 mM L-Glutamate to block body wall muscle contraction [21]. Motor neuron terminals were labeled with a ten to fifteen minute incubation in a rabbit primary antibody to HRP (Jackson ImmunoResearch) conjugated with Alexa-647 (Molecular Probes/Invitrogen) or goat primary anti-HRP pre-conjugated with DyLight 649 (Jackson ImmunoResearch) at a concentration of approximately 1 ug/50 ul of HL-6, followed by a 30 second rinse in HL-6. Diffusion barriers at the NMJ were tested by infusing the tissue with 10 kDa dextran conjugated with Alexa-647 (Molecular Probes/Invitrogen) dissolved in HL6 (with 2 mM Ca^2+^ and 5 mM Glutamate) at a concentration of 1 mg/1.5 ml [3].

### Fluorescence Labeling of Fixed Tissue

For tissue fixed for immunolabeling, feeding third instar larvae were dissected in HL-6 with 2 mM Ca^2+^ with 4% paraformaldehyde in everted or flat-pinned preps. To test “standard” fixation conditions phosphate buffered saline (PBS) without Ca^2+^ was used. Subsequent immunofluorescence labeling was carried out as previously described [30]. The following primary antibodies were used: anti-Dlg mAb 4F3 [60] at 1∶100 (NICHD, University of Iowa); anti-Repo mAb 8D12 [61] at 1∶50 (NICHD, University of Iowa); and anti-HRP rabbit polyclonal at 1∶300 (Jackson Labs). Secondary antibodies were used at 1∶300 and included goat anti-mouse or goat anti-rabbit conjugated to Alexa-488, Alexa-568 (Molecular Probes/Invitrogen) or Cy5 (AbCam). Tissue was equilibrated in 90% glycerol and mounted using Vectashield (Vector Labs).

### Microscopy

For live tissue image acquisition, we used two spinning disc confocal microscopes: the Quorum Wave FX systems and the Perkin Elmer UltraView VoX. Volocity software (PerkinElmer) was used with both systems for three dimensional image acquisition, deconvolution and video production. Deconvolution was carried out to an approximately 99% confidence level using calculated point spread functions. We collected images stacks, sampled at 0.2 µm Z spacing, with a 63X water immersion lens (NA 1.2) using an immersion oil of refractive index 1.3379 (Cargille Labs) matched to the refractive index of HL-6. We also collected images with a DeltaVision Spectris restoration system (Applied Precision), using a 60X oil immersion lens (NA 1.4), with 0.2 µm Z section increments. The image stacks were subsequently deconvolved to a residual value of 0.2 or less with SoftWorx (API), based on measured point spread functions of 0.1 µm (Figures 4 A; Video S4) or 0.2 µm fluorescent beads (Molecular Probes) mounted in Vectashield. Subsequent image analysis of fixed tissue with ImageJ was the same as for live tissue image stacks. Images for publication were processed and compiled with Photoshop CS4 (Adobe).

### Image Analysis and Morphometry

All images shown are from live synapses and muscles 6/7 in abdominal segment 2 (A2) unless otherwise noted. Since the glial processes were essentially flat, we measured the 2 dimensional area of GFP labeled glia including both synaptic lamella and small sections of non-synaptic motor axon branches from 2D projections. We also scored synapses as +/− for glial processes distal to the first synaptic bouton, as complementary test specificity to synaptic perineurial glial processes. We measured the 2D area of the SSR using the linear auto-contrast, auto-threshold, and area measurement functions of NIH ImageJ [62]. Similar measures were used for neuronal areas determined by anti-HRP immunolabeling and were collected for those neuronal regions distal to the axon and found only in the boutons.

For statistical analysis, we analyzed the synaptic regions of muscles 6/7 of individual larvae at abdominal segments A2 and A3, and pooled the data from both segments. Each two-dimensional area from each NMJ at muscles 6/7 constituted an independent replicate (“n”) value. Detailed statistical results are reported in Table 1. Statistical analysis was done with Prism (Graphpad Software) and ExcelStat. We aimed to assess two different features of glial projections: the size of the projections, and whether or not the treatment affected the likelihood of a glial extension. To measure significant differences in 2-D area means, at a 95% confidence interval, we used paired or unpaired t-tests (Figure 7); Mann-Whitney tests with Dunn’s post-test (fixed vs. live tissue); or one- or two way ANOVAs with Bonferonni’s Multiple Comparison post-test (Figures 6, 8). The same live wild-type data set, reared at three temperatures, and at stages F3 and W3 (Figure 7) served as controls and a statistical comparison group for fixation, drug treatment, and genetic mutations. We used Fisher’s exact tests, Chi-square tests and Binary Logistic Regression, as appropriate, to evaluate the likelihood (frequency) of a glial process being present at any synapse, as a function of rearing temperature, drug treatment, and genetic background.

## Supporting Information

Figure S1 **Glial processes within the larval NMJ are actin rich.** repo-GAL4 was used to drive GFP-tagged actin (green) and the postsynaptic regions were immunolabeling with Dlg (magenta) in fixed F3 larvae. Fixation conditions specifically incooporated artificial hemolymph HL-6 supplemented with Ca^2+^. Each panel represents a projection of a Z stack that was viewed enface or rotated on the Y-axis (ROT) to show the degree of process association with individual boutons. The exception is panel F, which was rotated on the X axis to show the “hollow” glial process (arrowhead). In the panels, examples of glial processes that associate with the labeled bouton (arrow) or have projections independent of the boutons (arrowheads) are shown. Scale bars are 15 microns in all panels.
(TIF)

Figure S2 **Glial processes at wandering 3rd instar NMJs were relatively static.** A–B) A live NMJ from a wandering third instar (W3) larvae with glial processes labeled using 46F::CD8GFP (green), neurons immunolabeled with anti-HRP (red) and the SSR labeled with ShCter-DsRed (blue). The NMJ was held at 25°C and imaged at time 0 (T0) and 60 minutes later (T60). Glial area at T0 = 11.63 µm^2^. Net change in glial area  = +0.52 µm^2^. Scale bar, 15 microns. C–J) Boxed regions from panels A and B were digitally scaled 400% with the corresponding grayscale panel showing the range of GFP tagged glial processes. Glial processes associated with boutons (C, D; arrows); and grew independently of either boutons or SSR (C, D: arrowhead). Glial processes appeared to change shape (E, F; arrow) and position with respect to the NMJ (G, H; arrow). Other processes retracted near the synapse (H, I; arrow) or remained associated with the immunolabeled neuron (H, I; arrowhead).
(TIF)

Figure S3 **Glial processes are found at the NMJ by the 2nd larval instar.** Glial processes labeled with CD8GFP in the 2nd larval instar. A–D) 46F-GAL4 driven CD8GFP expression (green) was detected within 2nd instar NMJs fixed and immunolabeled with anti-HRP (red) and anti-Dlg (blue) to label the pre- and post-synpatic regions of the NMJ respectively. At this stage the glial processes have extended into the synpatic region (arrow). Scale bar is 15 microns. Panels B–D were digitally scaled 400% (E–F) repo-GAL4 driven CD8GFP expression (green) imaged in live and intact 2nd instar larvae through the body wall. The subsynaptic reticulum labeled with ShCter-dsRed (magenta) indicates the location of the NMJ. At this stage the glial processes have extended into the synaptic region (F, arrow) and also show extra-synaptic extensions across the body wall muscle (E, arrow)
(TIF)

Video S1 **3D rotation over 90**° **of a live 3rd instar NMJ showing the glial processes labeled with 46F>mCD8GFP (green) along the central region of the synaptic bouton region.** α-HRP (red) was used to live immunolabel the presynpatic boutons and ShCter-DsRed (blue) labeled the postsynaptic SSR.
(MOV)

Video S2 **Manipulatable rotation around a 90**° **axis along the live 3rd instar NMJ shown in** **Figure 3** **.** The glial processes (green) were live labeled using 46F>CD8GFP, the neurons labeling using live α-HRP (red) immunolabeling and the SSR using ShCter-DsRed (blue).
(MOV)

Video S3 **Video panning through the Z-series collected from the 3rd instar NMJ shown in** **Figure 3** **.** The glial processes (green) were live labeled using 46F>CD8GFP, the neurons labeling using live α-HRP (red) immunolabeling and the SSR using ShCter-DsRed (blue). The pan starts in the more superficial image focal planes (nearest the viscera) (Figure 3D) and continues to the site where the motor axons enter between the muscles from the cuticular side (Figure 3G).
(MOV)

Video S4 **3D rotation over 90**° **of the fixed 3rd instar NMJ from** **Figure 5A** **.** The septate junction domain was labeled with Neurexin IV (green) along the motor axons as they branch across the NMJ. α-HRP (red) was used to immunolabel the presynpatic boutons and the postsynaptic SSR was immunolabeled for Dlg (blue).
(MOV)

Video S5 **Manipulatable rotation around a 90**° **axis along the live 3rd instar NMJ shown in** **Figure 8B** **.** The glial processes were live labeled with repo>CD8GFP (green) and boutons immunolabeled with α-HRP (magenta).
(MOV)
