# Supplementary figures and images for: Glial Processes at the Drosophila Larval Neuromuscular Junction Match Synaptic Growth
Source: PLoS One. 2012 May 29;7(5):e37876. doi: 10.1371/journal.pone.0037876 (PMC3362601; doi:10.1371/journal.pone.0037876)

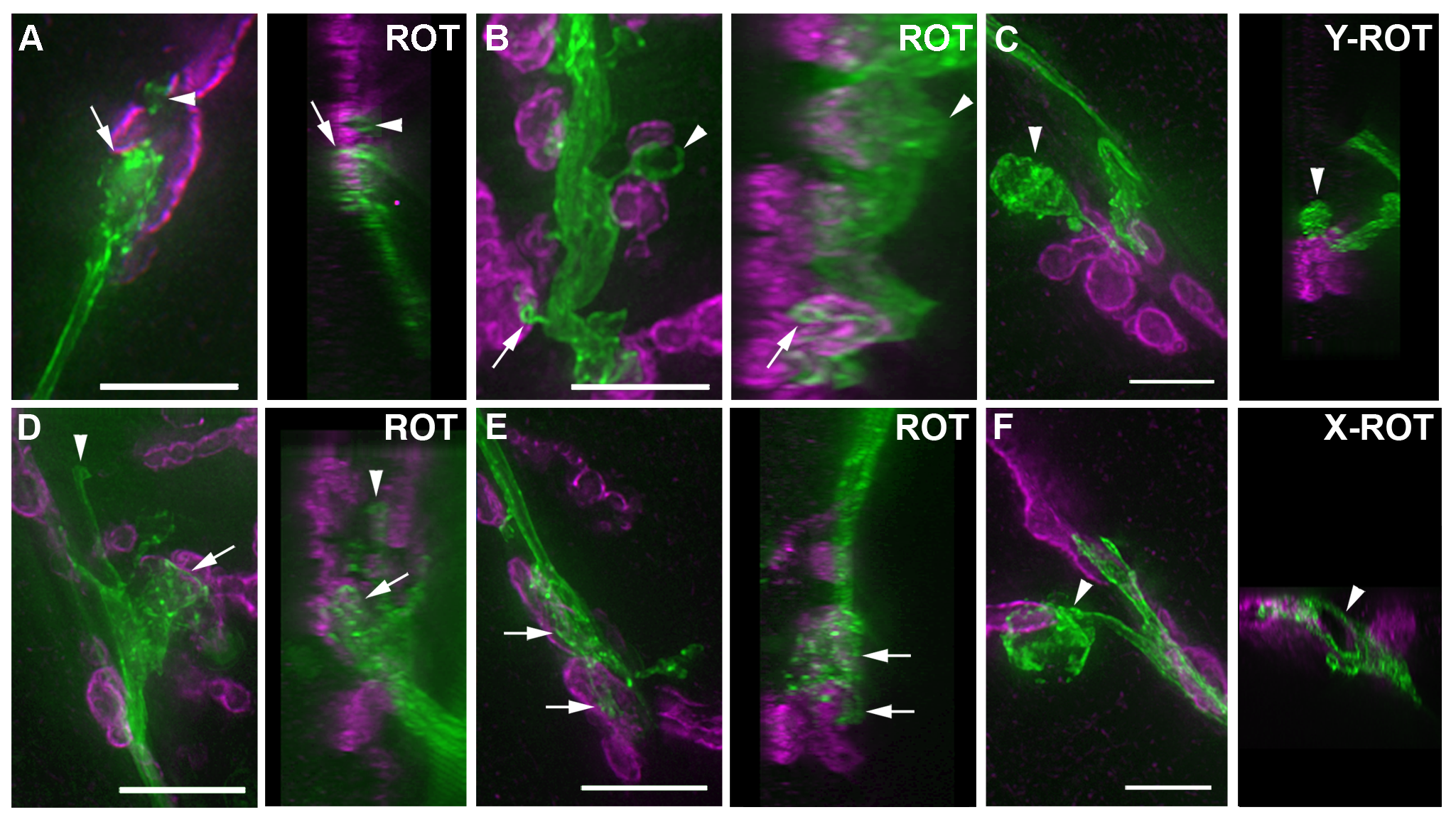

Supplement: Figure S1 — Glial processes within the larval NMJ are actin rich. repo-GAL4 was used to drive GFP-tagged actin (green) and the postsynaptic regions were immunolabeling with Dlg (magenta) in fixed F3 larvae. Fixation conditions specifically incooporated artificial hemolymph HL-6 supplemented with Ca2+. Each panel represents a projection of a Z stack that was viewed enface or rotated on the Y-axis (ROT) to show the degree of process association with individual boutons. The exception is panel F, which was rotated on the X axis to show the “hollow” glial process (arrowhead). In the panels, examples of glial processes that associate with the labeled bouton (arrow) or have projections independent of the boutons (arrowheads) are shown. Scale bars are 15 microns in all panels. (TIF) [file pone.0037876.s001.tif]

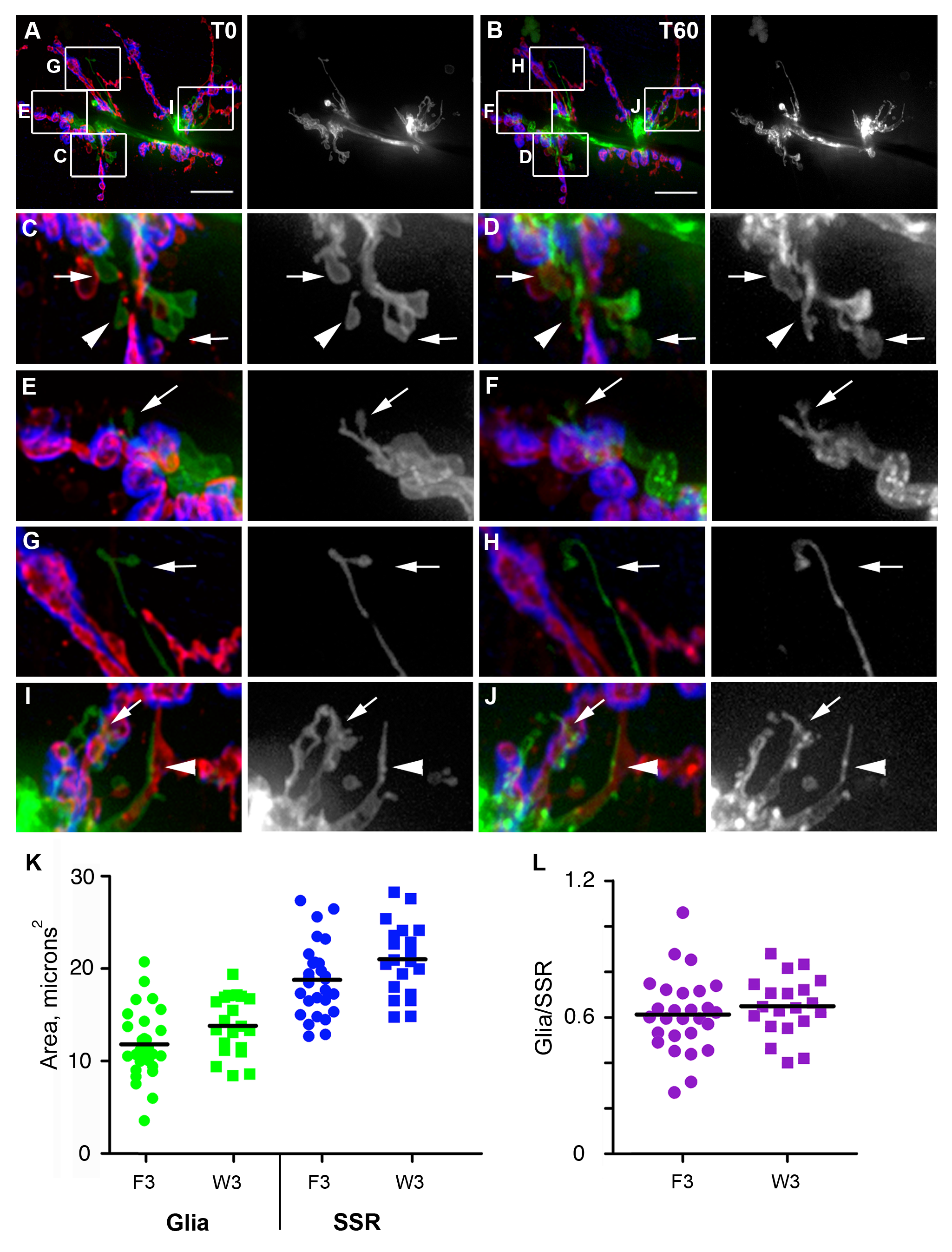

Supplement: Figure S2 — Glial processes at wandering 3rd instar NMJs were relatively static. A–B) A live NMJ from a wandering third instar (W3) larvae with glial processes labeled using 46F::CD8GFP (green), neurons immunolabeled with anti-HRP (red) and the SSR labeled with ShCter-DsRed (blue). The NMJ was held at 25°C and imaged at time 0 (T0) and 60 minutes later (T60). Glial area at T0 = 11.63 µm2. Net change in glial area = +0.52 µm2. Scale bar, 15 microns. C–J) Boxed regions from panels A and B were digitally scaled 400% with the corresponding grayscale panel showing the range of GFP tagged glial processes. Glial processes associated with boutons (C, D; arrows); and grew independently of either boutons or SSR (C, D: arrowhead). Glial processes appeared to change shape (E, F; arrow) and position with respect to the NMJ (G, H; arrow). Other processes retracted near the synapse (H, I; arrow) or remained associated with the immunolabeled neuron (H, I; arrowhead). (TIF) [file pone.0037876.s002.tif]

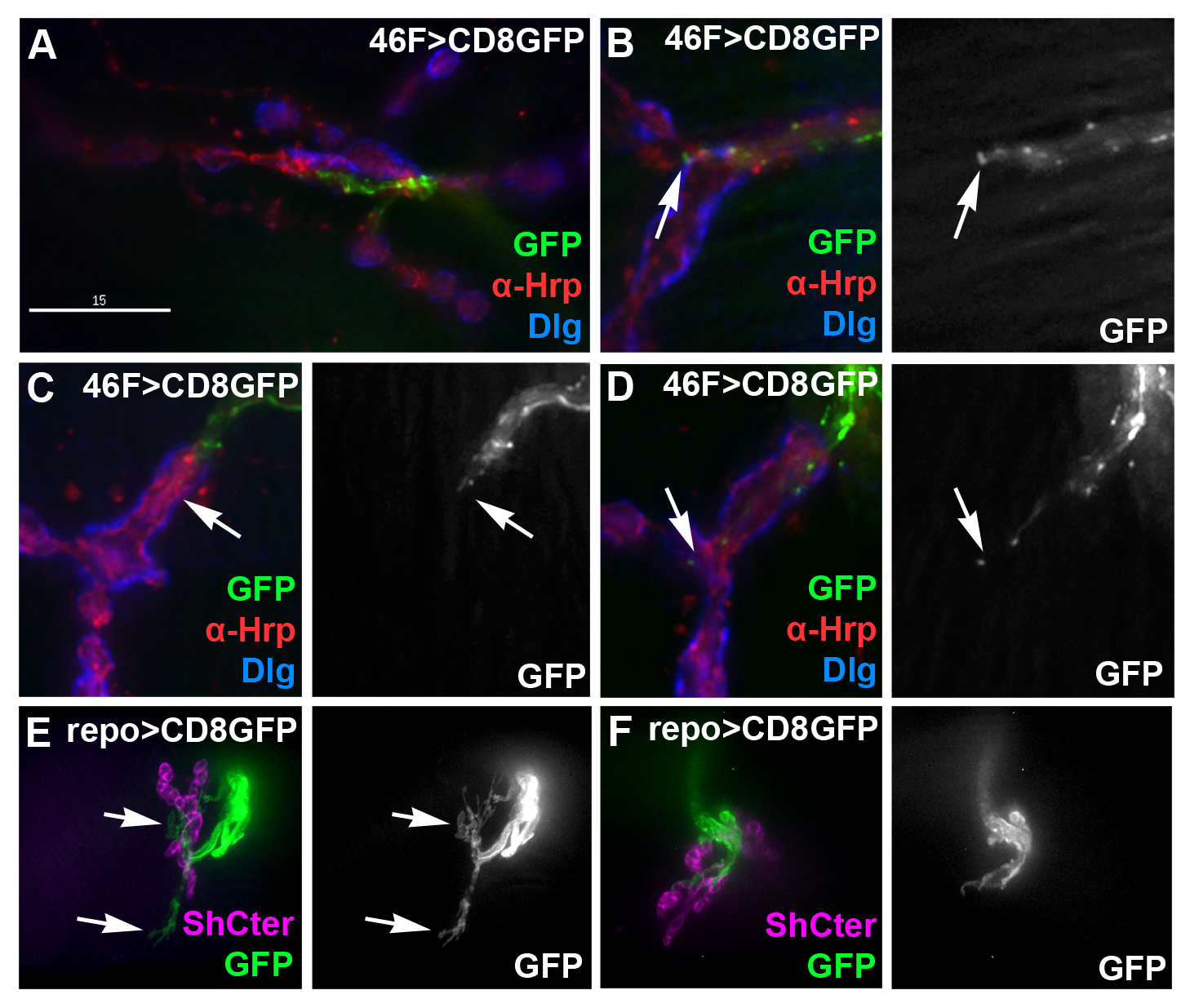

Supplement: Figure S3 — Glial processes are found at the NMJ by the 2nd larval instar. Glial processes labeled with CD8GFP in the 2nd larval instar. A–D) 46F-GAL4 driven CD8GFP expression (green) was detected within 2nd instar NMJs fixed and immunolabeled with anti-HRP (red) and anti-Dlg (blue) to label the pre- and post-synpatic regions of the NMJ respectively. At this stage the glial processes have extended into the synpatic region (arrow). Scale bar is 15 microns. Panels B–D were digitally scaled 400% (E–F) repo-GAL4 driven CD8GFP expression (green) imaged in live and intact 2nd instar larvae through the body wall. The subsynaptic reticulum labeled with ShCter-dsRed (magenta) indicates the location of the NMJ. At this stage the glial processes have extended into the synaptic region (F, arrow) and also show extra-synaptic extensions across the body wall muscle (E, arrow) (TIF) [file pone.0037876.s003.tif]
